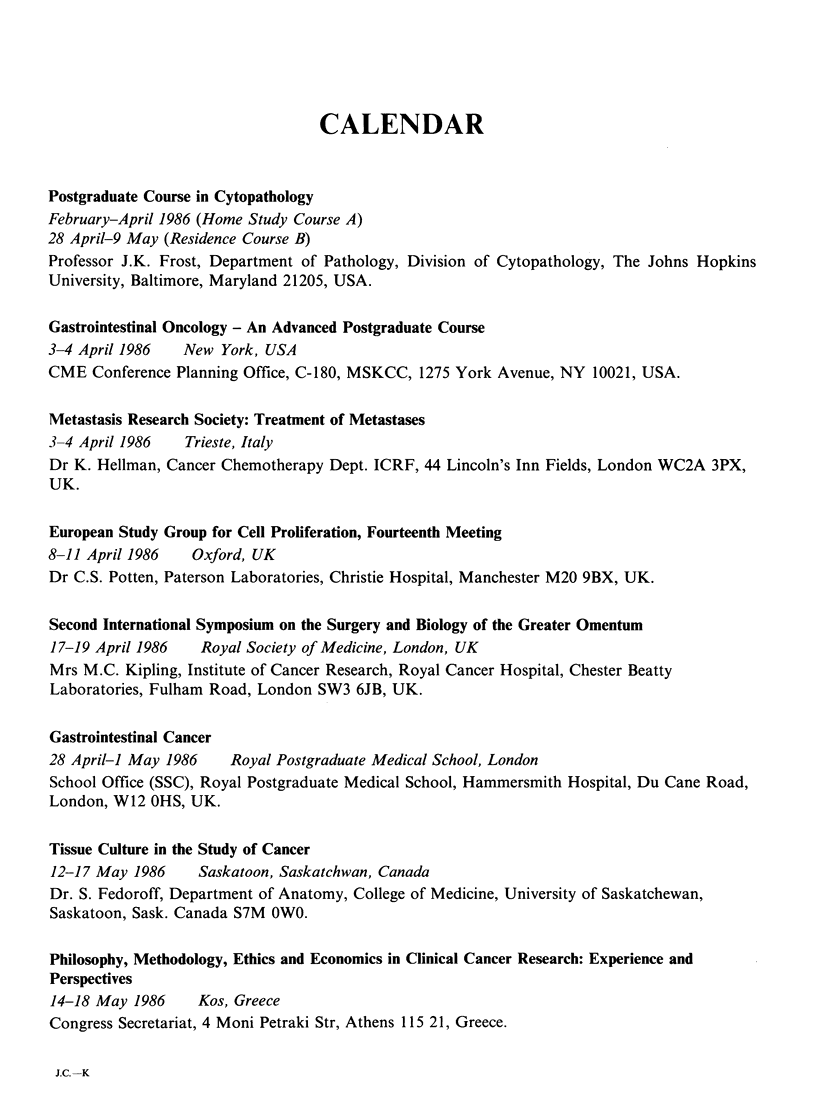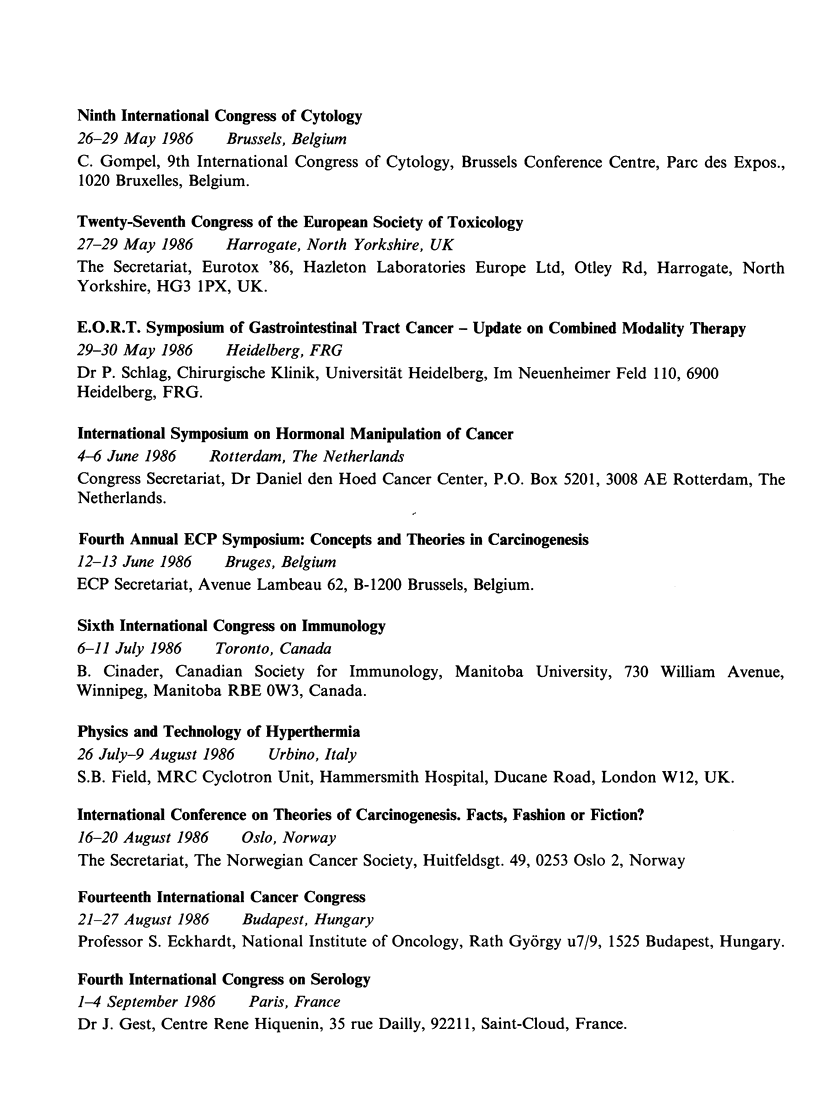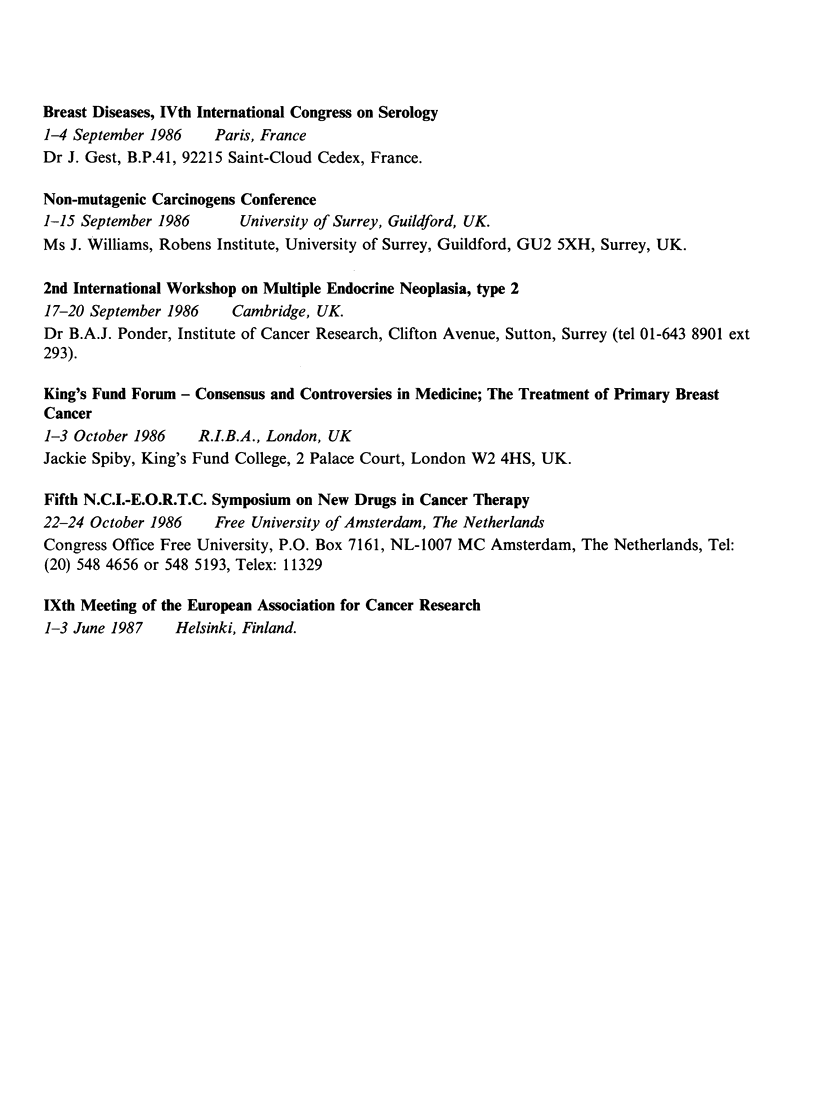# Calendar

**Published:** 1986-04

**Authors:** 


### CALENDAR

**Postgraduate Course in Cytopathology**

*February–April 1986 (Home Study Course A)*
*28 April–9 May (Residence Course B)*

Professor J.K. Frost, Department of Pathology, Division of Cytopathology, The Johns Hopkins University, Baltimore, Maryland 21205, USA.

**Gastrointestinal Oncology – An Advanced Postgraduate Course**

*3–4 April 1986    New York, USA*

CME Conference Planning Office, C-180, MSKCC, 1275 York Avenue, NY 10021, USA.

**Metastasis Research Society: Treatment of Metastases**

*3–4 April 1986    Trieste, Italy*

Dr K. Hellman, Cancer Chemotherapy Dept. ICRF, 44 Lincoln's Inn Fields, London WC2A 3PX, UK.

**European Study Group for Cell Proliferation, Fourteenth Meeting**

*8–11 April 1986    Oxford, UK*

Dr C.S. Potten, Paterson Laboratories, Christie Hospital, Manchester M20 9BX, UK.

**Second International Symposium on the Surgery and Biology of the Greater Omentum**

*17–19 April 1986    Royal Society of Medicine, London, UK*

Mrs M.C. Kipling, Institute of Cancer Research, Royal Cancer Hospital, Chester Beatty Laboratories, Fulham Road, London SW3 6JB, UK.

**Gastrointestinal Cancer**

*28 April–1 May 1986    Royal Postgraduate Medical School, London*

School Office (SSC), Royal Postgraduate Medical School, Hammersmith Hospital, Du Cane Road, London, W12 0HS, UK.

**Tissue Culture in the Study of Cancer**

*12–17 May 1986    Saskatoon, Saskatchwan, Canada*

Dr. S. Fedoroff, Department of Anatomy, College of Medicine, University of Saskatchewan, Saskatoon, Sask. Canada S7M 0W0.

**Philosophy, Methodology, Ethics and Economics in Clinical Cancer Research: Experience and Perspectives**

*14–18 May 1986    Kos, Greece*

Congress Secretariat, 4 Moni Petraki Str, Athens 115 21, Greece.

**Ninth International Congress of Cytology**

*26–29 May 1986 Brussels, Belgium*

C. Gompel, 9th International Congress of Cytology, Brussels Conference Centre, Parc des Expos., 1020 Bruxelles, Belgium.

**Twenty-Seventh Congress of the European Society of Toxicology**

*27–29 May 1986 Harrogate, North Yorkshire, UK*

The Secretariat, Eurotox '86, Hazleton Laboratories Europe Ltd, Otley Rd, Harrogate, North Yorkshire, HG3 1PX, UK.

**E.O.R.T. Symposium of Gastrointestinal Tract Cancer – Update on Combined Modality Therapy**

*29–30 May 1986 Heidelberg, FRG*

Dr P. Schlag, Chirurgische Klinik, Universität Heidelberg, Im Neuenheimer Feld 110, 6900 Heidelberg, FRG.

**International Symposium on Hormonal Manipulation of Cancer**

*4–6 June 1986 Rotterdam, The Netherlands*

Congress Secretariat, Dr Daniel den Hoed Cancer Center, P.O. Box 5201, 3008 AE Rotterdam, The Netherlands.

**Fourth Annual ECP Symposium: Concepts and Theories in Carcinogenesis**

*12–13 June 1986 Bruges, Belgium*

ECP Secretariat, Avenue Lambeau 62, B-1200 Brussels, Belgium.

**Sixth International Congress on Immunology**

*6–11 July 1986 Toronto, Canada*

B. Cinader, Canadian Society for Immunology, Manitoba University, 730 William Avenue, Winnipeg, Manitoba RBE 0W3, Canada.

**Physics and Technology of Hyperthermia**

*26 July–9 August 1986 Urbino, Italy*

S.B. Field, MRC Cyclotron Unit, Hammersmith Hospital, Ducane Road, London W12, UK.

**International Conference on Theories of Carcinogenesis. Facts, Fashion or Fiction?**

*16–20 August 1986 Oslo, Norway*

The Secretariat, The Norwegian Cancer Society, Huitfeldsgt. 49, 0253 Oslo 2, Norway

**Fourteenth International Cancer Congress**

*21–27 August 1986 Budapest, Hungary*

Professor S. Eckhardt, National Institute of Oncology, Rath György u7/9, 1525 Budapest, Hungary.

**Fourth International Congress on Serology**

*1–4 September 1986 Paris, France*

Dr J. Gest, Centre Rene Hiquenin, 35 rue Dailly, 92211, Saint-Cloud, France.

**Breast Diseases, IVth International Congress on Serology**

*1–4 September 1986 Paris, France*

Dr J. Gest, B.P.41, 92215 Saint-Cloud Cedex, France.

**Non-mutagenic Carcinogens Conference**

*1–15 September 1986 University of Surrey, Guildford, UK.*

Ms J. Williams, Robens Institute, University of Surrey, Guildford, GU2 5XH, Surrey, UK.

**2nd International Workshop on Multiple Endocrine Neoplasia, type 2**

*17–20 September 1986 Cambridge, UK.*

Dr B.A.J. Ponder, Institute of Cancer Research, Clifton Avenue, Sutton, Surrey (tel 01-643 8901 ext 293).

**King's Fund Forum – Consensus and Controversies in Medicine; The Treatment of Primary Breast Cancer**

*1–3 October 1986 R.I.B.A., London, UK*

Jackie Spiby, King's Fund College, 2 Palace Court, London W2 4HS, UK.

**Fifth N.C.I.-E.O.R.T.C. Symposium on New Drugs in Cancer Therapy**

*22–24 October 1986 Free University of Amsterdam, The Netherlands*

Congress Office Free University, P.O. Box 7161, NL-1007 MC Amsterdam, The Netherlands, Tel: (20) 548 4656 or 548 5193, Telex: 11329

**IXth Meeting of the European Association for Cancer Research**

*1–3 June 1987 Helsinki, Finland.*